# Unveiling the Hidden Treasury: CIITA-Driven MHC Class II Expression in Tumor Cells to Dig up the Relevant Repertoire of Tumor Antigens for Optimal Stimulation of Tumor Specific CD4+ T Helper Cells

**DOI:** 10.3390/cancers12113181

**Published:** 2020-10-29

**Authors:** Greta Forlani, Mariam Shallak, Fabrizio Celesti, Roberto S. Accolla

**Affiliations:** Laboratories of General Pathology and Immunology “Giovanna Tosi”, Department of Medicine and Surgery, University of Insubria, 21100 Varese, Italy; greta.forlani@uninsubria.it (G.F.); mshallak@uninsubria.it (M.S.); fcelesti1@uninsubria.it (F.C.)

**Keywords:** CD4+ T helper cells, CIITA, MHC class II, tumor vaccines, combination therapy

## Abstract

**Simple Summary:**

It is becoming clear that combined approaches are required to fight and succeed against cancer. As far as immune-based strategies, new generation anti-tumor vaccines will certainly play a crucial role. A key aspect is to identify tumor antigens which optimally stimulate CD4+ T helper cells, as these cells are crucial to triggering and maintaining all effector mechanisms of the adaptive immune response. Our approach is to render tumor cells surrogate antigen presenting cells for their own tumor antigen by forcing them to express de novo MHC class II molecules after the genetic transfer of CIITA, the major transcriptional controller of MHC class II gene expression. The unprecedented strong stimulation of tumor-specific CD4+ T cells that were obtained with our approach let us hope that this will help in identifying a constellation of MHC class II-restricted tumor antigens for more potent next generation anti-tumor vaccines.

**Abstract:**

Despite the recent enthusiasm generated by novel immunotherapeutic approaches against cancer based on immune checkpoint inhibitors, it becomes increasingly clear that single immune-based strategies are not sufficient to defeat the various forms and types of tumors. Within this frame, novel vaccination strategies that are based on optimal stimulation of the key cell governing adaptive immunity, the CD4+ T helper cell, will certainly help in constructing more efficient treatments. In this review, we will focus on this aspect, mainly describing our past and recent contributions that, starting with a rather unorthodox approach, have ended up with the proposition of a new idea for making available an unprecedented extended repertoire of tumor antigens, both in quantitative and qualitative terms, to tumor-specific CD4+ T helper cells. Our approach is based on rendering the very same tumor cells antigen presenting cells for their own tumor antigens by gene transfer of CIITA, the major transcriptional coordinator of MHC class II expression discovered in our laboratory. CIITA-driven MHC class II-expressing tumor cells optimally stimulate in vivo tumor specific MHC class II-restricted CD4 T cells generating specific and long lasting protective immunity against the tumor. We will discuss the mechanism underlying protection and elaborate not only on the applicability of this approach for novel vaccination strategies amenable to clinical setting, but also on the consequence of our discoveries on sedimented immunological dogmas that are related to antigen presentation.

## 1. Introduction

**Figure A1:**
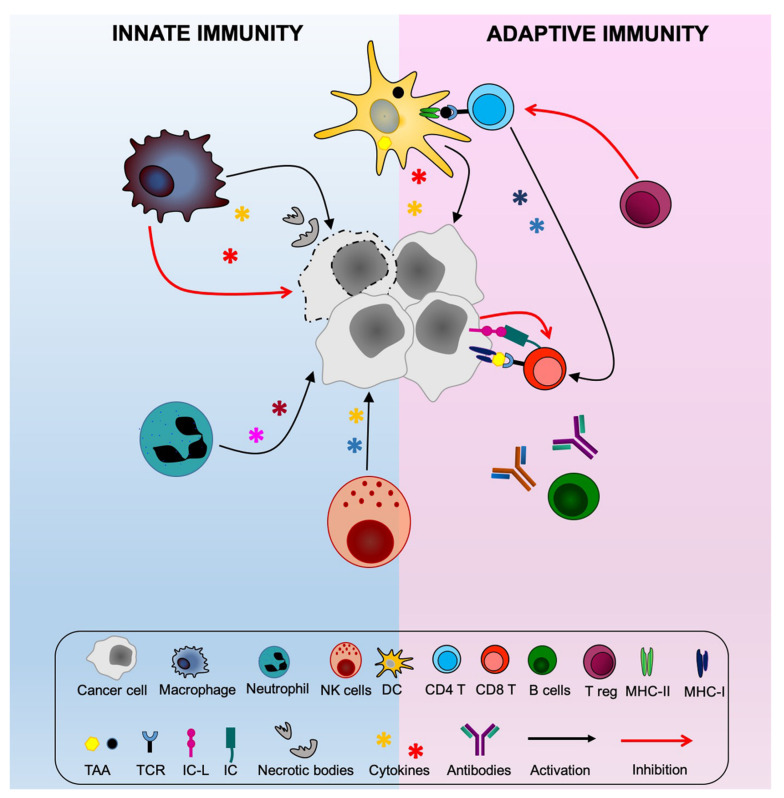
The basic elements of anti-tumor immunity. Cells of the innate immunity such as macrophages and neutrophils, attracted at the tumor site, participate to the destruction and engulphment of the tumor cells via secretion of several products such as inflammatory cytokines (black arrows). Tumor cells may polarize the phenotype of macrophages toward a pro-tumor phenotype (red arrows) by modifying their production of inflammatory cytokines in anti-inflammatory cytokines. At the edge of innate and adaptive immunity, NK cells participate to the host immune response by killing tumor cells that have lost MHC class I expression to elude adaptive CTL immunity. Dendritic cells engulphing necrotic tumor cell debris process and present tumor antigens via their MHC class II molecules to CD4+ Th cells which then trigger the effector mechanisms of anti-tumor immunity, such as antibody producing B cells and particularly CD8+ CTL (black arrows), by secreting specific cytokines. CD4+ Th activity can be blocked by regulatory T cells (Treg), thus hampering the activation of downstream CTL. Moreover, tumor cells can directly block the action of CTL by expressing immune checkpoints ligands (IC-L), such as PD-L1, via interaction with PD-1 (IC) expressed on CTL.

The immune response against cancer, or better against cancers, is a complex series of events that are regulated by both intrinsic and extrinsic mechanisms, whose final goal should be the elimination of the tumor cells or at least the control of their growth [1]. However, in this long lasting fight, tumor cells do not remain neutral or passive. In fact, they try to elude the attack of the immune system, particularly of the adaptive arm of immunity, basically in two ways: by hiding themselves from the recognition of tumor-specific lymphocytes, and by creating and orchestrating an immunosuppressive microenvironment that inhibits, blocks, or even kills immune effectors [2,3,4]. The process of active elusion from specific immune recognition is rather paradoxical, because it shares with the neoplastic transformation the common denominator of alteration of the cellular genome [5]. While structural mutations and/or disregulations of gene expression that are at the basis of oncogenesis should contribute to create neo-antigens or overexpressed cellular proteins that are recognizable by the immune system, in the same time they might affect genes that are strongly involved in antigen presentation such as major histocompatibility complex (MHC) molecules, in particular, MHC class I (MHC-I) molecules [6]. These molecules, designated HLA-A, -B, -C in human, are normally expressed in all cell types. Their function consists in binding and displaying on the cell surface endogenously derived antigenic peptides, loaded in the endoplasmic reticulum (ER), for scrutiny by MHC-restricted CD8+ T lymphocytes with cytolytic function (cytotoxic T lymphocytes or CTL) [7]. CD8+ CTL are believed to be the major effectors of the adaptive anti-tumor response (Figure A1). Recognition by CTL is mediated by a clonotypically distributed receptor (T cell receptor or TCR) ontogenically selected to recognize non-self antigens. Because tumor antigens, due to mutations or overexpression, behave as non-self antigens, they could be recognized by CTL [8] and, thus, tumor cells expressing them could be easily eliminated. The lack of expression of MHC-I molecules in tumor cells may thus represent an efficient way to elude recognition by CTL [6].

However, the triggering, proliferation, and maintenance of tumor-specific CTL strongly depend on the activity of another T cell family, broadly defined as T helper (Th) cell subpopulation [9,10,11] (Figure A1). These cells share the same ontogenetic origin of the CTL, as they functionally mature in the thymus, they similarly express clonotypically distributed TCR and they recognize antigens only when presented by MHC molecules. Nevertheless, significant differences from CTL cells are present in Th cells, as they express the lineage marker CD4 and they recognize mostly, but not always as we will see, exogenously derived antigenic peptides presented by specialized cells through their MHC class II (MHC-II) molecules (HLA-DR, DP, and DQ in human) [7].

MHC-II molecules expression is limited to only few cell types, mainly dendritic cells (DC), macrophages and B cells, defined as professional antigen-presenting cells (APC) [12,13]. This definition stems from the fact that APC, particularly DC and macrophages, are equipped not only with MHC-II molecules, but also with a repertoire of molecules and intracellular organelles particularly suited for digesting and processing endocytosed materials [13]. Indeed, at variance with MHC-I, loading of peptides on MHC-II molecules preferentially takes place in acidic endosomal compartments [14], where degraded products from endocytosed external materials are particularly concentrated and where MHC-II molecules, complexed with the invariant (In) chain, a molecule that prevents premature peptide loading in endoplasmic reticulum, are routed after biosynthesis [15,16]. Because of these features, it is widely assumed that MHC-II molecules cannot present peptides derived from the processing of endogenously synthesized molecules. The generalization of this assumption, however, has been challenged by many elegant studies showing that endogenous proteins could access the MHC-II pathway of antigen presentation [17,18,19], and peptides of these proteins could be recognized and serve as immunogens for Th cell triggering [20,21].

Tumor cells, particularly carcinoma cells, do not express MHC class II molecules and apparently do not share phenotypic features of classical APC; thus, they do not have the possibility to stimulate Th cells and, consequently, to initiate the cascade of event leading to anti-tumor effector functions. The inability of tumor cells to trigger Th cells has contributed to substantiating the immunological dogma, as shown for a wide variety of antigens, including pathogens, that tumor antigens could trigger the Th response only if endocytosed, processed, and presented by professional APC [13,22]. Consequently, this would limit the processing and presentation of tumor antigens to tumor cell debris and possibly secreted tumor cell products that APC can capture in the tumor microenvironment. In this condition, the potential repertoire of tumor antigens that professional APC could process and expose via their MHC-II molecules for CD4+ Th cell scrutiny is relatively limited in both quality and quantity.

## 2. Exploring New Avenues for Optimal Stimulation of Anti-Tumor CD4+ Th Cells

On the basis of the above considerations, it was initially thought that suitable approaches to rescue the adaptive immune response against the tumor should concentrate on the activation of those effector cells capable to directly eliminate the tumor cells, i.e., the CTL [8]. Several vaccination and immune therapeutic approaches based on MHC-I-bound tumor antigen characterization and on amplification of preselected antigen-specific CTL, respectively, were investigated. The results were poor, particularly when promising protocols that were tested in the experimental animal were transferred to clinical setting [23]. Beside the already described down-regulation of MHC-I expression in tumor cells in vivo hampering CD8+ CTL recognition, it is now well established that insufficient response was due to the lack of efficient help provided to CTL by CD4+ Th cells [24,25]. Moreover, even if tumor vaccination strategies have more recently revitalized the interest toward the stimulation of tumor-specific Th cells [26,27], the limitation remains regarding the biochemical nature and number of tumor antigens that can be offered to these cells in vivo and their optimal stimulation once they see the antigen.

Our group tried to approach the above fundamental issues in a rather unorthodox way, taking in consideration the knowledge on the intimate mechanism of MHC-II gene expression and the described evidence that endogenous antigens may be presented by MHC-II molecules [17,18,20]. Thus, we investigated whether tumor cells may act as surrogate APC of their own tumor antigens, provided that they could be modified to express MHC-II molecules in a “physiological” modality.

## 3. The CIITA Approach

In previous publications, we have extensively reviewed our approach aimed at rendering tumor cells constitutively positive for MHC class II expression by the genetic transfer of the MHC class II transactivator [24,25,28,29,30] discovered in our laboratory [31,32,33,34], also designated CIITA [35]. Here, we will briefly review our data and frame them within the context of the crucial role of CD4+ Th cells.

The MHC class II transactivator was initially discovered by our group by studying isogenic mutants of the Burkitt lymphoma cell line Raji [36]. Genetic complementation studies subsequently showed that CIITA was acting across species barriers and coordinated the expression of entire family of MHC class II genes [31,32]. Moreover, and importantly, CIITA controlled the expression of DM genes necessary for a correct loading of peptides in the MHC class II loading compartment [37] and upregulated the expression of In chain [38] that, as described above, acts as chaperone for MHC-II heterodimers driving them to the peptide loading compartment, where In chain detaches from MHC-II and allows peptide loading. Constitutive and inducible MHC-II gene expression, like the one mediated by interferon gamma (IFNɣ), are both under the control of CIITA, thus showing the physiological crucial role of CIITA in coordinating the machinery of antigen presentation [39]. Therefore, it was tempting to investigate whether the forced expression of CIITA into tumor cells could render these cells more “visible” and, thus, immunogenic for tumor specific CD4+ Th cells. We were encouraged in this belief by preliminary experiments showing that MHC-II-negative human hepatocarcinoma cell lines stably expressing transfected CIITA became MHC-II positive and could process and present antigenic peptides to antigen-specific primed Th cells [40]. The same was subsequently shown to also be valid for mouse tumor cell lines of distinct histotype, [41]. Indeed, when applied to in vivo experimental animal models of highly tumorogenic MHC-II-negative cancer cells we could demonstrate that CIITA-induced MHC-II expression was instrumental in rendering these tumor cells immunogenic and rejectable, or strongly retarded in their growth, when injected in syngeneic immunocompetent recipients including mammary, colon and renal carcinomas, as well as sarcomas [42,43]. Other investigators have tried to render tumor cells more immunogenic by transfecting isolated MHC alpha and beta chain-encoding genes [44,45]. By only expressing isolated MHC class II molecules without expression of In chain, both the district of interaction and quality of interacting peptides, including tumor-associated peptides, are totally different when compared to the site and the peptides interacting with physiologically expressed MHC class II molecules. The rationale underlying this approach was to allow peptides from tumor antigens, which are endogenous proteins, to associate with MHC class II molecules in the ER, similarly to what happens for MHC class I-peptide binding, and thus allow better recognition of putative tumor antigens by MHC class II-restricted CD4+ Th cells. However, although protective immunity could be generated in vivo by vaccinating mice with alpha-beta MHC-II-transfected cells, the cellular correlates of protection remained incompletely clarified, because, essentially, a single tumor model was studied in that approach, specifically the SaI sarcoma. It was also suggested that tumor cells may not directly act as surrogate APCs, but as donors of peptide-MHC class II complexes for professional APC, such as DC, which, in turn, stimulate Th cells [45]. However, it must be kept in mind that, in the absence of In chain, hardly any MHC class II molecules are in a stable peptide-loaded form. Cells from In chain knock-out mice show a dramatic reduction in cell surface MHC class II molecules, resulting from both defective association of class II alpha/beta chains and markedly decreased post-Golgi transport. The few class II alpha/beta heterodimers reaching the cell surface behave as empty molecules or as molecules that are occupied by an easily displaced peptide, and they display a distinct structure. Moreover spleen cells from these mice are defective in their ability to present intact protein antigens [46,47]. Our model, instead, was based on the “physiological” expression of MHC-II molecules and, consequently, on the idea of classical MHC-II-restricted presentation of tumor antigen peptides to CD4+ Th cells. Thus, it was important to establish the relative contribution of distinct cellular subpopulation to the in vivo protection against CIITA-driven MHC-II expressing tumor cells in our model.

## 4. Optimal Stimulation of Tumor-Specific CD4+ Th Cells by CIITA-Driven MHC Class II Expressing Cells

Initially, in the mouse mammary carcinoma TSA model, the in vivo depletion of distinct lymphocytes subpopulations before injecting CIITA-expressing tumor cells was performed. The results clearly indicated that the depletion of either naive CD4+ or naïve CD8+ T cells, but not B cells or NK cells, abrogated the protection against live CIITA-tumor cells [42]. These results were for us a clear suggestion that CD4+ T cells were key in triggering a protective MHC class II-restricted anti-tumor immune response, because parental tumor cells were not capable to induce a similar protective CD4 or CD8 immune response. It remained to be established the mechanism through which MHC class II-restricted CD4+ T cells would act in vivo to protect the animals from tumor growth.

In a first series of experiments, it was found that the in vivo depletion of CD4+ T cells in CIITA-tumor vaccinated and protected mice did not dramatically alter the capacity of vaccinated mice to respond and reject parental-tumor cells. On the contrary, in vivo depletion of CD8+ T cells drastically reduced the immune response of vaccinated mice to parental tumors, resulting in tumor growth. This gave support to the idea that CD4+ Th cells were crucial in inducing the anti-tumor immune response, but not in acting as direct effectors against the tumor cells, in a similar way to CD8+ CTL. The in vivo depletion experiments were then further complemented by adoptive cell transfer approaches using purified immune CD4+ or CD8+ T cells. To clearly define the relative mechanism of in vivo action of the distinct immune T cell subpopulations, we set up a rather articulated protocol. First, we transferred immune CD4+ or CD8+ T cells into naïve syngeneic recipients and assessed the capacity of these cells to protect the mice from parental tumor challenge. Interestingly, we found that immune anti-tumor CD4+ T cells were equally, and, in many cases, even more potent than immune anti-tumor CD8+ T cells in protecting naïve animals from parental tumor take and growth. This feature was common to several tumor models of distinct histotype, including the mammary TSA, the colon C51 and the renal Renca carcinomas, and the WEHI sarcoma [43,48]. Thus, although immune CD4+ T cells might not act as direct effectors in eliminating the tumor cells, they were absolutely necessary and sufficient for protecting naïve animals from very tumorogenic tumors. How to reconcile the apparent dichotomy of CD4+ T cells being necessary to trigger and maintain the in vivo immune response against the tumor and yet being almost dispensable in vaccinated immune mice to reject a challenge with parental tumor cells? The issue was partially clarified by subsequent experiments, in which we adoptively transferred the same immune CD4+ T cells into naïve recipients that were previously depleted of CD8+ T cells. The results showed that primed immune CD4+ T cells could not re-establish full protection from challenge with CIITA-negative parental tumor, strongly suggesting that primed anti-tumor CD4+ Th cells could trigger and activate tumor-specific naïve CD8+ T cells to make them fully mature anti-tumor CTL effectors [43]

**Figure 1:**
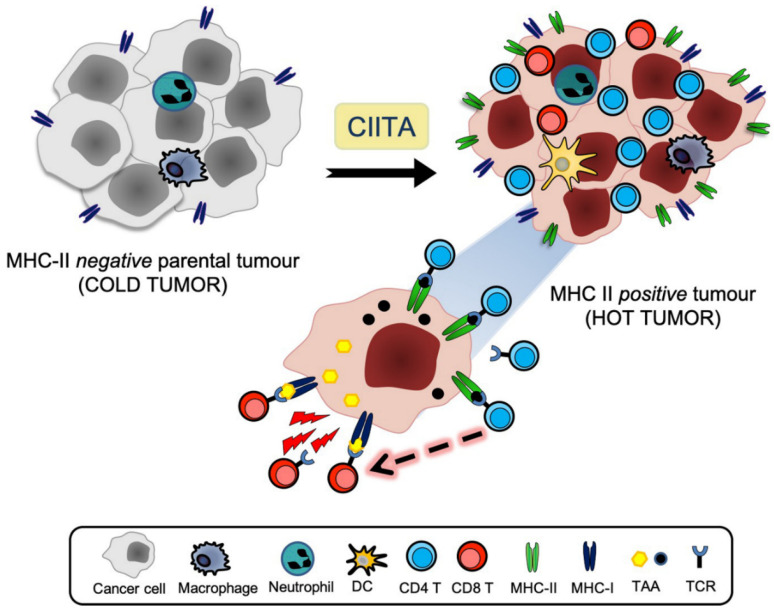
CIITA-driven MHC-II+ tumor cells are immunogenic and promote a drastic change in the tumor microenvironment Expression of CIITA-driven MHC-II expression in tumor cells drastically modify the histology of the tumor microenvironment. Parental tumor cells are very little infiltrated by blood-derived cells (cold tumor); upon stable transfection with CIITA and consequent expression of MHC class II molecule, tumors became rapidly infiltrated by CD4+ T cells, followed by CD8+ T cells (hot tumor). An increased tumor antigen repertoire is offered to MHC-II-restricted CD4+ Th cells, which, once activated, sustain the activation of specific antitumor CD8+ T cells.

Taken together, these studies gave strong support to the real helper nature of CD4+ T cells in initiating and supporting the anti-tumor immune response, provided that they were triggered by CIITA-driven MHC-II+ tumor cells. Once generated, these anti-tumor TH cells could be maintained in vivo for long time, ready to help triggering of MHC-I-restricted CD8+ CTL when mice were challenged with parental tumors whose MHC class I-tumor antigen complexes could be seen by specific T cell receptors that were expressed on CD8+ T cells [43,48] (Figure 1). Here, definitely the presence and availability in the tumor microenvironment of sufficient amounts of key cytokines, such as IL-2 and IFNɣ produced by anti-tumor primed CD4+ T cells, facilitated the activation and function of CD8+ CTL effectors [41,42,43,48]. Whether a direct action of tumor-specific CD4+ Th cells as cytotoxic effectors, clearly proven in several animal models [49,50], and recently also shown in human tumors by single cell sequencing [51,52], may also partially operate in our model cannot be totally excluded, although it certainly does not represent a concomitant major mechanism.

## 5. The Tumor Microenvironment and the APC Function of CIITA-Driven MHC-II-Expressing Tumor Cells

The absolute requirement of CIITA-driven MHC-II positive tumor cells to trigger tumor-specific CD4+ Th cells and initiate the cascade of events, leading to an efficient adaptive anti-tumor immune response raised several key questions. For example, could a specific correlate of protection be shown in vivo in the tumor tissue and, importantly, could CIITA-tumor cells function as APC to directly prime tumor–specific virgin CD4+ Th cells in vivo?

**Figure 2:**
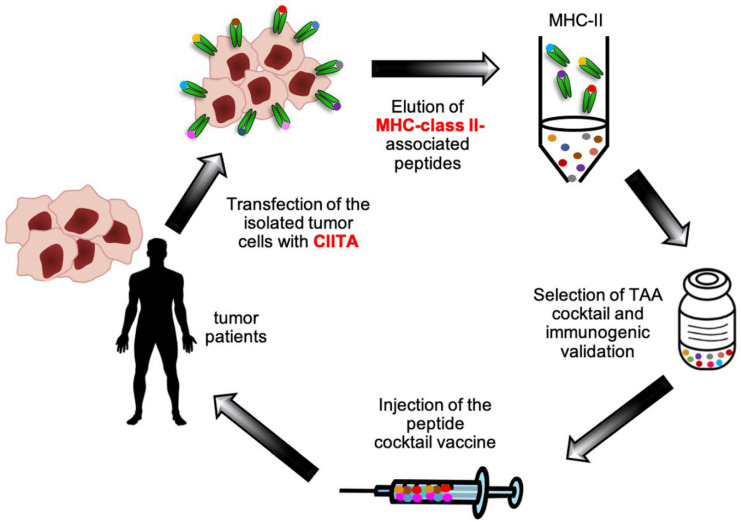
The forced expression of CIITA in tumor cells paves the way for the construction of MHC-II based multi-peptide anticancer vaccine. MHC-II negative tumor cells isolated form patients are genetically modified by forcing the expression of CIITA and consequently the expression of MHC class II genes. The neo-expressed MHC-II-peptide complexes are purified, peptides eluted and sequenced for the construction of MHC-II restricted multi-peptide vaccine cocktail. Upon immunogenic validation, the vaccine is injected in tumor patients.

To answer the first question, we performed kinetic studies of tumor rejection in the TSA tumor model after injection of CIITA-tumor cells, in conjunction with immunohistology of the tumor tissue. Interestingly, it was found that CIITA-tumor tissue was rapidly infiltrated by CD4+ T cells already at day 4 after tumor cell injection. CD4+ T cell infiltration was followed 3–6 days later by DC and CD8+ T cell infiltration. This was in clear contrast with the tumor tissue that was generated by parental tumor cells, which was virtually free of infiltrating linfo-monocyte cells during its growth [41,43]. These findings were strongly suggestive of a direct function of CIITA-driven MHC-II expressing tumor cells as APC in vivo. However, there were not still conclusive for a direct priming of virgin tumor specific CD4+ Th cells, as the possibility existed that MHC-II-tumor antigen complexes shed by CIITA-tumor cells or derived by apoptotic/necrotic tumor cells and captured by DC, could be used by DC to complement their otherwise insufficient APC function in lymph nodes, by a mechanism of cross-dressing [45,53] or by the recently described intracellular vesicle exchange between distinct subpopulation of DCs [54]. The crucial issue was mostly solved by the use of an experimental mouse model that was based on the transgenic strain CD11c.DTR on the C57BL/6 H-2^b^ genetic background. CD11c.DTR transgenic mice express the diphtheria toxin receptor (DTR) downstream the CD11c promoter which is highly active in DC [55]. Treatment with diphtheria toxin (DT), induces long-lasting depletion of DCs thus permitting the assessment of the direct priming of naïve CD4+ T cells by MHC-II-expressing tumor cells. When these mice were injected with CIITA-driven MHC-II-expressing tumor cells, here we used the MC38 colon carcinoma and the LLC Lewis Lung carcinoma, after conditional depletion of DCs, they were still capable of rejecting tumor cells or strongly retard their growth, clearly showing that CIITA-driven MHC-II-expressing tumor cells were the most important surrogate APC in vivo for their own tumor antigens [56]. Moreover, the depletion of macrophages, the other important APC in vivo was similarly ineffective in reducing the immune response against CIITA-tumor cells, giving further support to the notion that CIITA-driven MHC class II expressing tumor cells were the relevant in vivo APC to prime CD4+ Th cells [56]. These results have a dual value, as they challenge the widely accepted dogma that DC are the only cell type performing in vivo priming of MHC-II-restricted CD4+ Th cells [13,57] and, at the same time, offer the opportunity to investigate the repertoire of potential tumor antigens expressed by these modified tumor cells for novel vaccination strategies [29,30] (Figure 2). Moreover, in consideration of the fact that in our experimental model CD4+ T cells are the first to infiltrate the CIITA-driven MHC-II positive cells in vivo, it is very likely that the initial priming of tumor specific CD4+ Th cells may take place in the tumor tissue, possibly in ectopic lymphoid aggregations outside the canonical lymphoid tissues. These structures are referred to as tertiary lymphoid structures (TLS) or ectopic lymphoid-like structures and they have been observed in inflamed and, more interestingly, in tumor tissues [58,59]. TLS display several features of lymph nodes that are associated with the generation of an adaptive immune response [59,60].

## 6. From Bench to Bedside: New Hope for the Future of Immmunotherapy?

Is the adequate antigen availability [28] to prime and stimulate naïve CD4+ Th cells generated by CIITA-driven MHC-II expressing tumor cells useful in clinical setting for novel strategies of anti-tumor therapy? A preliminary attempt has been made recently by a European Consortium (the Hepavac Consortium, www.hepavac.eu), made of nine distinct laboratories and research centers that focused their action on the construction of a multipeptide vaccine against hepatocarcinoma. A series of both MHC-I-restricted (HLA-A2 and HLA-A24) and MHC-II-restricted peptides directly purified from hepatocarcinomas were selected following the procedure and purification platform originally described by Rammensee and coll [61]. Because hepatocarcinoma cells, like their normal tissue counterpart, do not express MHC-II molecules because lack of CIITA expression [62], they were genetically induced by transfection of CIITA, the neo-expressed HLA-DR-peptide complexes purified, peptides eluted and sequenced. From 6629 peptides screened, four were selected based on their specific expression only on hepatocarcinomas and not in normal hepatocytes or in other tumors of distinct histotype. The vaccine cocktail included 11 similarly selected HLA class I-restricted peptides. Immunogenicity data are currently evaluated and will soon be available. We are now trying to apply the same strategy to assess the MHC-II immunopeptidome of CIITA-modified human glioblastomas, the most malignant tumor of the central nervous system, whose prognosis is dismal (less than 10% survival after five years from diagnosis) and for which new therapeutic approaches are urgently needed.

## 7. Conclusions

**Figure 3:**
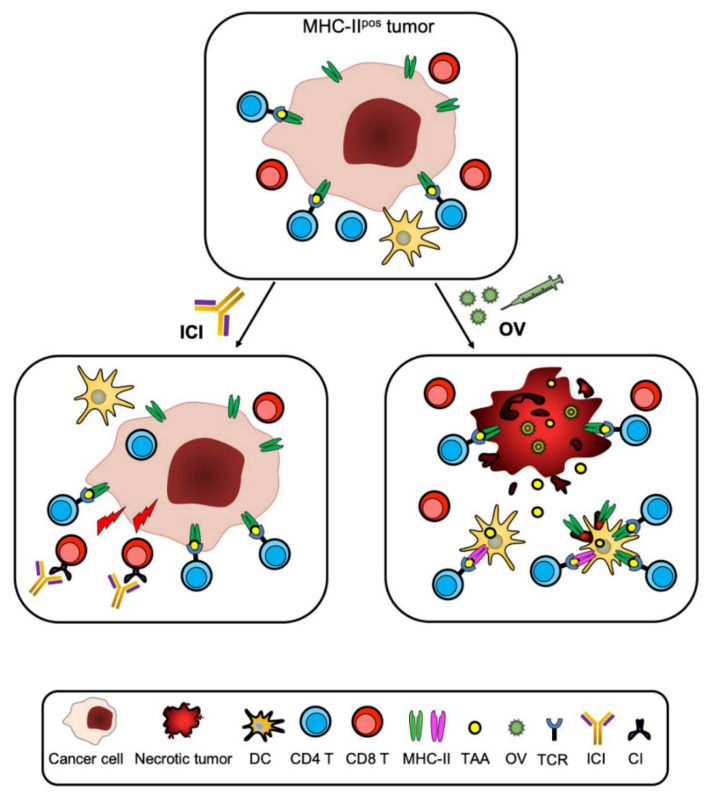
Increasing the breath and efficacy of anti-tumor immunotherapy by combined approaches. CIITA-driven MHC-II expression in tumor cells can be combined to additional immunotherapeutic strategies to increase tumor antigen recognition and effector cell activation. For example, CD4+ Th cells triggered by MHC-II+ tumor cells can activate anti-tumor CTL and synergize with inhibitory anti-immune checkpoint mAbs (ICI) in rescuing exhausted anti-tumor CTL, thus potently increasing both the number and cytotoxic activity of CTL. Furthermore, tumor antigen availability generated by CIITA-driven MHC-II expressing tumor cells can be increased by the action of oncolytic viruses (OV) that upon killing of the tumor cells will liberate additional antigenic material either free or under the form of MHC-II-peptide complexes derived from CIITA-expressing tumor cells. The combined antigen-presenting cell (APC) function of MHC-II-expressing cells and classical professional APCs infiltrating the tumor will increase the capacity of tumor-specific CD4+ Th cells to recognize tumor antigens and initiate the cascade of events leading to effective anti-tumor immunity.

Approaches of cancer immunotherapy have recently been boosted by the discovery that immune checkpoint-specific monoclonal antibodies (ICmAbs), such as antibodies against CTLA-4, PD-1, and PD-L1, can rescue the adaptive cellular immune response, mostly CTL, kept at bay by the tumor [63,64]. Justified enthusiasm has been however mitigated by the fact that many patients do not respond to ICmAbs and/or do eventually relapse or manifest adverse effects [65]. At present, the idea that effective anticancer therapy must therefore be based on combined approaches that synergize to eliminate tumor mass is increasingly taking the stage. Based on these observations, we may hypothesize that combining our approach with ICmAb treatment and/or with other promising immune-related strategies such as oncolytic virus (OV) treatment [66,67], may increase both the rescue and breath of adaptive immune response against the tumor, further modifying what is generally called a “cold” immune unresponsive tumor microenvironment to a “hot” immunologically active and responsive tumor microenvironment [57] (Figure 1 and Figure 3). Within this frame, recent advances in engineering bispecific antibodies targeting CD28 costimulatory molecule should also be considered for optimizing cancer-specific immunotherapy [68]. In our view, combined approaches that include our strategy could offer additional therapeutical opportunities to patients with malignancies that are not amenable to other classical therapies, giving new hope for the cure of their disease.
